# A rare cause of neonatal hypercalcemia: Neonatal severe primary hyperparathyroidism: A case report and review of the literature

**DOI:** 10.1016/j.ijscr.2019.12.024

**Published:** 2019-12-28

**Authors:** Tural Abdullayev, Mevlit Korkmaz, Mustafa Kul, Nuray Koray

**Affiliations:** a Department of Pediatric Surgery, Medical Park Gebze Hospital, Güzeller, Kavak Cd. No: 5, 41400, Gebze, Kocaeli, Turkey; b Department of Pediatric Surgery, EMSEY Hospital, Çamlık Mah. Selçuklu Cad. No: 22 Pendik, İstanbul, Turkey; c Departments of Neonatal İntensive Care Unit, Emsey Hospital, Çamlık Mah. Selçuklu Cad. No: 22 Pendik, Istanbul, Turkey; d Departments of General Surgery, Private Korfez Marmara Hospital, Güney, Albay Sk. No: 7, 41780, Körfez, Kocaeli, Turkey

**Keywords:** Hypercalcemia of the newborn, Neonatal hyperparathyroidism, CASR gene mutation, Intraoperative PTH monitoring, Sestamibi scan

## Abstract

**Introduction:**

Neonatal severe primary hyperthyroidism is an extremely rare disorder that occurs in the first six months of life. Early recognition and prompt surgical intervention are of vital importance for survival and to avoid neurological sequel. Hypotonia, lethargy, respiratory distress, and growth and developmental delay occur in association with elevated serum parathormone levels and hypercalcemia (Gannon et al., 2014). Definitive therapy involves total parathyroidectomy.

**Case presentation:**

We are presenting a patient with Neonatal severe primary hyperparathyroidism, who successfully underwent total parathyroidectomy. The patient had been followed up with medical therapy until he was seven months old, with no adequate clinical response to medical therapy. Parathormone levels rapidly declined following total parathyroidectomy, and the parathormone level fell to zero after removal of the ectopic tissue with a second surgery, and the patient was discharged with full recovery.

**Discussion:**

Sestamibi scintigraphy might not always show an ectopic parathyroid gland. In such conditions, confirmation of parathyroid glands excised with total parathyroidectomy by frozen biopsy is not sufficient to terminate surgery. Intraoperative parathormone monitoring is particularly important at this point. Persistently elevated parathormone levels should suggest a remnant parathyroid tissue at the surgical site or an ectopic parathyroid gland that needs to be excised.

**Conclusion:**

Neonatal severe primary hyperparathyroidism is a life-threatening condition. Early surgery is life-saving in cases in whom medical therapy fails to control the disease.

## Highlights

• Neonatal severe primary hyperparathyroidism is an exceedingly rare condition that has high mortality and morbidity if left untreated.
• Medical therapy must be initiated as soon as the condition is diagnosed, and early surgery must be performed in patients who are refractory to the medical therapy.
• Scintigraphic studies might sometimes fail to detect ectopic parathyroid glands. Intraoperative parathormone monitoring is particularly important to ensure complete removal of the parathyroid glands.

## 1 Introduction

Neonatal severe primary hyperparathyroidism is an extremely rare condition that manifests with severe hypercalcemia and metabolic bone disease within the first few weeks in the postnatal period. Growth and developmental delay, hypotonia, polyuria, dehydration, gastrointestinal dysmotility, poor feeding, and respiratory distress are common symptoms. Emergency surgery must be performed if medical therapy fails to stabilize the clinical condition of the patient [1]. The current report presents an infant boy with neonatal hyperparathyroidism and discusses the approach to diagnosis and treatment. The work has been reported in line with the SCARE criteria [2]. The informed consent was obtained from the patient’s father for publication of this case report.

## 2 Case presentation

A 7-month-old male infant was admitted to our clinic with the diagnosis of neonatal severe primary hyperparathyroidism. It was understood from the medical history that the patient was born full-term to a 41-year-old mother via normal vaginal route with a weight of 2.900 g and height of 51 cm, mother and father are consanguineous (cousins), the patient had been hospitalized multiple times due to hypercalcemia, the patient had been diagnosed with neonatal hyperparathyroidism based on the laboratory tests and genetic analysis, the patient had sustained septic episodes multiple times due to prolonged hospitalizations and that surgery was postponed until the patient weighted 5 kg. Blood samples had been previously collected from the parents for genetic tests and both were found to have familial hypocalciuric hypercalcemia (two copies of the missense mutation pro39ala in both parents).

**Fig. 1:**
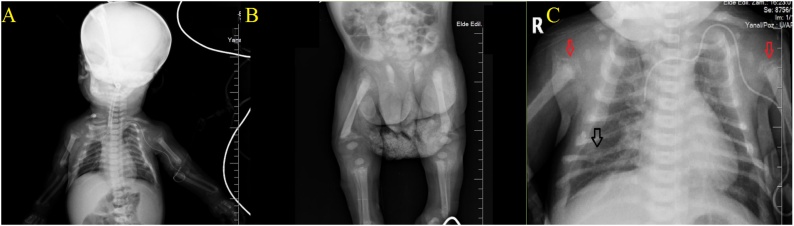
A. An atelectatic area in the right lung. B. Demineralization defects in the lower extremities. C. Bell-shaped chest cavity, rib fracture (black arrow), mineralization disorder in humeral epiphyses.

The weight of the patient was 4.300 g, and the height was 62 cm on admission. He had severe respiratory distress and subcostal retractions. Lung sounds were bilaterally diminished on auscultation, and there were rales and rhonchi. Chest x-ray revealed rib fracture, pneumonic infiltrations in both lungs and an atelectatic area at the apex of the right lung. The patient was intubated and admitted to the intensive care unit. The whole-body x-ray revealed osteopenia and impaired mineralization of periosteal areas (Fig. 1).

**Fig. 2:**
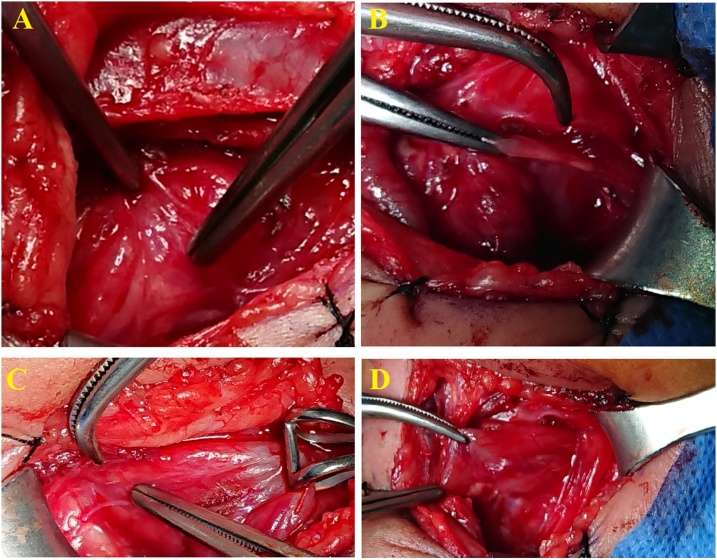
All parathyroid glands. A. Right upper pole parathyroid gland. B. Right lower pole parathyroid gland. C. Left upper pole parathyroid gland. D. Left lower pole parathyroid gland.

**Fig. 3:**
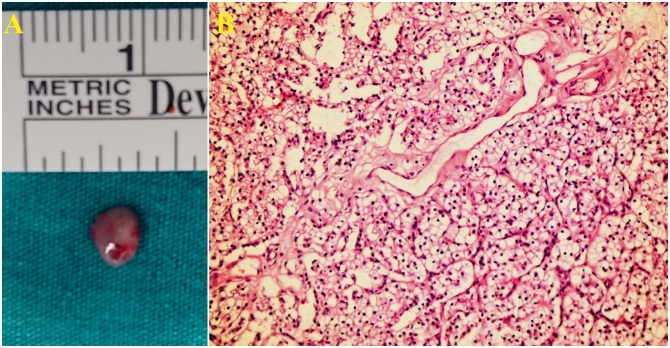
A. Gross appearance of hyperplasic right upper pole parathyroid gland. B. Microscopic examination of this gland shows parathyroid cell hyperplasia.

The patient was on a therapy with phosphate Sandoz and cinacalcet which he did not regularly receive due to his deteriorating clinical condition in the last few days; laboratory tests showed parathormone level of 1.835 pg/ml, calcium was 17.89 mg/dl, alkaline phosphatase was elevated (586 U/L) and magnesium (1.36 mg/dL) and phosphorous (1.74 mg/dL) were decreased due to increased bone turnover. Intravenous therapy involving fluids, furosemide, pamidronate, and cinacalcet was commenced. The patient was extubated after two weeks and underwent surgery while he was placed in an oxygen hood. Total parathyroidectomy was performed (Fig. 2). The right upper parathyroid gland had a more hyperplasic appearance than the other glands (Fig. 3). All parathyroid glands were excised and sent to the laboratory for frozen examination, since intraoperative parathormone monitoring was not available at our clinic. The operation was terminated upon confirming by the frozen examination that all the four parathyroid glands have been successfully removed. Recurrent laryngeal nerve injury, hemorrhage, and infection did not occur in the postoperative period.

**Fig. 4:**
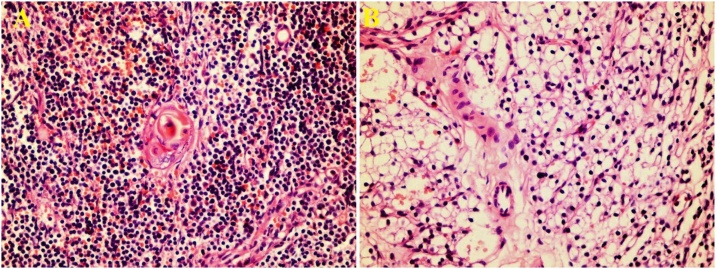
A. Thymus, B. Ectopic Parathyroid Gland inside the Thymus Gland.

The parathormone level was found to be 615 pg/mL in blood samples obtained after surgery, and thus cinacalcet therapy was resumed. The elevated parathormone level was considered to be related to an ectopic tissue. However, repeat sestamibi scintigraphy did not reveal an ectopic tissue. A decision was made to perform a second surgical operation. We evaluated the surgical area in detail again during the second operation. However, we could not find any parathyroid gland. We decided to evaluate different ectopic localizations in which we could not show ectopic gland on sestamibi scintigraphy. However, we have not been able to find the parathyroid gland even though we have repeatedly examined these localizations. In such a case, we had two options. Either look at the posterior mediastinum by thoracoscopy or excise the most common site of the ectopic gland and wait for the response to frozen examination. Since the parathyroid gland in the posterior mediastinum is very difficult to be detected by thoracoscopy in infants and will significantly prolong the surgical period, the second option was applied considering the unstable status of the patient. The patient underwent partial left lower pole thyroidectomy (the part associated with the thyrothymic ligament) and excision of thyroid thyrothymic extension. However, when the parathyroid gland could not be seen on frozen examination, it was decided to remove the thymus gland. The ectopic parathyroid gland was very small and was found embedded in the thymus tissue on frozen examination (ectopic and supernumerary intrathymicparathyroid gland) (Fig. 4). The parathormone level was 8 pg/mL on postoperative day one, and calcium was 4.49 mg/dL. Emergency calcium replacement therapy was initiated. The parathormone level has fallen to zero in routine blood tests obtained on postoperative day three.

The patient was disconnected from the respirator on day five after the second surgery. However, his oxygen demand continued after extubation due to his chronic disease. The patient was discharged with oral replacement therapy [alfacalcidol and calcium].

## 3 Discussion

Neonatal severe primary hyperparathyroidism is a rare condition characterized by severe hypercalcemia and metabolic bone disease that manifests in the first six months of life and more often in the first weeks of life. Growth and developmental delay are often accompanied by hypotonia, polyuria, and dehydration. Gastrointestinal dysmotility, poor feeding, and respiratory distress are other symptoms. Severe bone demineralization, bell-shaped chest deformity, osteopenia, subperiosteal bone resorption, and bone fractures are characteristic radiographic findings. Laboratory studies reveal increased serum calcium and parathormone levels, hypocalciuria, hypophosphatemia due to decreased tubular phosphorous resorption and hyperphosphaturia [1].

**Fig. 5:**
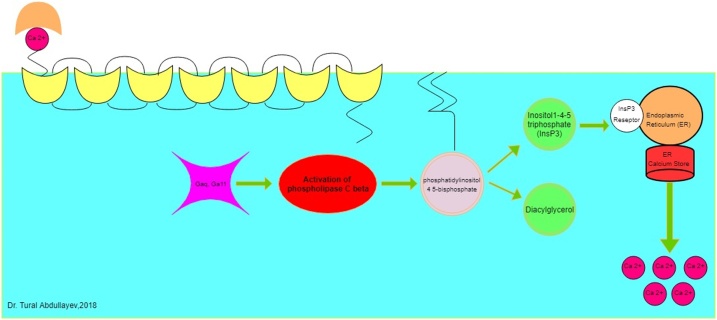
CaSR and calcium interaction.

The disease has a genetic basis showing autosomal recessive inheritance in which extracellular calcium does not inhibit parathormone release due to an inactivating mutation in calcium-sensing receptor (CaSR) gene. The primary function of the CaSR gene is to maintain calcium homeostasis by regulating calcium absorption from the gastrointestinal tract, calcium excretion from the urinary tract, and bone formation and resorption (Fig. 5). The CaSR is expressed in the thyroid and parathyroid glands, and it induces the release of parathormone from the parathyroid gland or calcitonin release from the C cells in the thyroid gland. Apart from these, CaSR is also found in the kidney epithelium, bones (osteoblasts, osteocytes, and bone marrow), and the intestine. These receptors are also involved in fibroblast proliferation, induction of cellular differentiation in keratinocytes and colon epithelium, inhibit apoptosis of prostate carcinoma cells, and regulate the development of cataract in lens epithelial cells [3,4].

The gene responsible for the CaSR mutations is located on chromosome 3q 13.3–21, and more than 300 mutations have been reported to date in the literature [http://www.casrdb.mcgill.ca] [5]. The mutations in the CaSR gene can be homozygous or heterozygous [6]. Homozygous mutations result in severe hypercalcemia that manifests early in the neonatal period [neonatal severe primary hyperparathyroidism], whereas heterozygous mutations result in a mild disease course characterized by asymptomatic hypercalcemia (Familial Hypocalciuric Hypercalcemia, FHH) [7]. In the etiology of FHH, mutations in two other genes, in addition to the CaSR gene, have been found to play a role in disease etiology (CaSR mutations in 60%, GNA mutations in 5%, AP2S1 mutations in 20%). The conditions have been named as FHH1, FHH2, and FHH3, in respective order, because the mutations in these genes result in the same clinical appearance and similar syndromes. Rarely, antibodies emerging against calcium-sensing receptors may also cause FHH [8,9].

Imaging methods used in the diagnosis of neonatal hyperparathyroidism are sestamibi scintigraphy (MIBI), ultrasound, and magnetic resonance imaging (MRI). Among the imaging methods, sestamibi scintigraphy is the most sensitive (85–88%) and specific (95–97%) to the parathyroid gland. Sestamibi scintigraphy is highly sensitive in demonstrating parathyroid glands in ectopic localizations. However, in the literature review, some publications reported failure in preoperative diagnosis with diagnostic tests [10].

Treatment options include medical and surgical therapies. Medical therapy might provide a complete cure in selected patients depending on the CaSR genotype [10]. Commonly used medications in medical therapy include intravenous fluids, abundant hydration, loop diuretics, calcitonin, intravenous bisphosphonates (pamidronate), and calcimimetic agents such as cinacalcet [11].

A treatment plan must be swiftly made to avoid irreversible complications in the long term, such as nephrocalcinosis, cardiac abnormalities [12], bone resorption or central nervous system changes, and emergency surgery must be performed if medical therapy fails to stabilize clinical and laboratory parameters. Although subtotal parathyroidectomy (3 and a half gland) has been previously regarded as a valid surgical approach, total parathyroidectomy has become the main surgical option afterward. There exist publications reporting the success of total thyroidectomy + autotransplantation in the treatment of neonatal hyperparathyroidism [1,13]. The rate of failure after grafting was reported to be 6%, and the rate of graft-associated hypercalcemia was reported to be 33% [14].

The localization of all parathyroid glands must be recognized during surgery, particular attention must be paid to avoid injury to the inferior and superior thyroid arteries supplying the thyroid gland, and injury to the recurrent laryngeal nerve must be avoided. Superior parathyroid glands are located at the level of cricoid cartilage 1 cm above the intersection of the inferior thyroid artery and recurrent laryngeal nerve, and inferior parathyroid glands are located posterolateral to the lower pole of the thyroid gland that is below the intersection of inferior thyroid artery and recurrent laryngeal nerve. Even mild bleeding must be avoided during surgery. Otherwise, isolation of the parathyroid glands will be complicated during bleeding or in the hemorrhagic operation site. Surgery is more challenging in newborns owing to limited surgical site, small parathyroid glands, and high likelihood of mistaking parathyroid glands with the lymph nodes. The use of intravenous methylene blue injection in conditions where the localization of the parathyroid glans is complicated is controversial. Although Al-Khalaf et al. reported the benefits of methylene blue injection during surgery, Janik et al. reported no benefits of doing so [15,16]. Blood parathormone level must be monitored during parathyroidectomy using intraoperative parathormone monitoring (IPM), the decision to continue exploration must be made accordingly, and the surgeon must be satisfied that sufficient amount of parathyroid gland has been excised [18,19]. The results of blood tests in samples sent during surgery become available within 20 min. As plasma half-life of parathormone is 3–5 min, the blood parathormone level must be monitored approximately 10 min after excision of the parathyroid glands, and the course of surgery must be planned accordingly. A significant decline in parathormone levels to the normal ranges implies that hyperfunctioning parathyroid glands have been sufficiently removed. The surgical site must be re-explored if no significant decrease occurs in parathormone levels and parathyroid glands in ectopic localizations must be explored. Some authors recommended bilateral jugular venous blood sampling during surgery and investigation of ectopic parathyroid glands at the side with elevated parathormone levels. Parathyroid glands left in the ectopic localizations will rapidly undergo hyperplasia and result in further release of parathormone [17]. Many published studies have implicated ectopic glands in the case of persistent hyperparathyroidism that is refractory to medical therapy [18]. Ectopic parathyroid glands may be located in the neck (thyrothymic ligament connecting the lower pole of the thyroid gland to the thymus), retropharyngeal area, intrathyroidal area, retroesophageal area, carotid sheet, and mediastinum (intrathymic and posterior mediastinum). The intrathyroidal ectopic parathyroid gland can be visualized by ultrasound that is performed by the surgeon during surgery. It is particularly important to make a differentiation between intrathymic and posterior mediastinal localizations in ectopic parathyroid glands that are found to be located in the mediastinum before surgery. A cervical incision will suffice in intrathymic parathyroid glands, whereas posterior mediastinal parathyroid glands require a thoracic approach. Histopathological examination of the excised parathyroid glands shows chief cell hyperplasia [19].

Inadequate parathyroidectomy or remaining parathyroid glands in ectopic localizations must be kept in mind in case of failed surgery and failure of parathormone levels to return to normal levels after surgery. Such conditions often necessitate a second surgery, but there are also studies reporting complete recovery with the use of bisphosphonate and a calcimimetic agent after failed surgery [20]. However, no clinical response was achieved in the present case to bisphosphonate and calcimimetic agents that were used with the anticipation of an ectopic gland, and thus surgery was planned afterward.

The most critical limitation in the present study is the lack of IPM. The use of IPM may have obviated the need for a second surgery. Although a frozen examination confirmed the removal of four parathyroid glands, IPM would show elevated parathormone levels and thereby direct the surgeon to an ectopic parathyroid gland. Sestamibi scintigraphy failed to show the ectopic gland, although the patient underwent this procedure twice, once before every surgical intervention. In such cases, the surgical site must be re-explored if IPM does not show a dramatic decrease in parathormone levels after removal of the parathyroid glands and the most common ectopic localizations of the parathyroid gland must be explored.

## 4 Conclusion

Neonatal severe primary hyperparathyroidism is a life-threatening condition. Medical therapy should not be delayed, and early surgery must be considered if the patient remains refractory to medical therapy. The procedure should not be terminated prematurely by only relying on the results of frozen examination during total parathyroidectomy, and intraoperative parathormone monitoring should definitely be performed. Although sestamibi scintigraphy is sensitive in demonstrating parathyroid glands in ectopic localization [19], persistent parathormone release in cases without a demonstrable ectopic gland on sestamibi scintigraphy should prompt re-exploration of the surgical site and performing further exploration for ectopic parathyroid glands.

## Funding

The case report had no sponsors.

## Ethical approval

This case report is exempt from ethical approval by our institution.

## Consent

The informed consent was obtained from the patient’s father for publication of this case report.

## Authors contributions

Tural Abdullayev, Mevlit Korkmaz and Nuray Koray operated the patient together. Mustafa Kul performed medical follow-up and treatment of the patient in the intensive care unit.

Tural Abdullayev MD: first author, contributed to the study concept, data collection, data analysis, and writing the paper; Mevlit Korkmaz MD: senior author and the manuscript reviewer, contributed to the study concept, data analysis, and manuscript; and Nuray Koray MD and Mustafa Kul MD reviewed the manuscript.

## Registration of research studies

This is not a ‘first in humans’ report, so it is not in need of registration.

## Guarantor

Mevlit Korkmaz.

## Provenance and peer review

Not commissioned, externally peer-reviewed

## Declaration of Competing Interest

No potential conflicts of interest.
